# The Sterilsation of the Unfit

**Published:** 1913-10-11

**Authors:** 


### THE STERILISATION OF THE UNFIT.

Though the medical profession may not be unanimous yet it will be admitted that a large body of medical opinion has declared that idiocy, insanity, imbecility, and feeble-mindedness are hereditary. With regard to criminality, opinion is not so definite and settled, but those who have investigated and classified the statistics of crime have arrived at a similar conclusion as to it also. These conclusions being admitted, assent may readily be given to the proposition that criminality and insanity, in their various phases, are conditions of life which should not be passed on. The good of the State demands that—*salus populi suprema lex*; and the sterilisation of those classes by the State has suggested itself as a means of preventing such continuance. To put that remedy into practice is a most drastic application of the Latin maxim quoted; it would be to take away from the individual the natural right to reproduce one's kind, a right as old as the human race, one which the object of the law hitherto has been to preserve; it would be a step on the road to the methods of Lycurgus, who caused the deformed babe to be torn from its mother's breast and conveyed to the wilderness for the wild animals to devour, to the end that all the sons of Sparta might be physically perfect. Yet in the United States of America, the persistence of the advocates of Eugenics, the plausibility of the doctrines which pass under that name, together with the efforts of the various State Boards of Health, have succeeded in obtaining legislation enacting the sterilisation of certain classes.

The statutes of five States—namely, California, Indiana, Connecticut, Iowa, and New Jersey—provide for the sterilisation of idiots, imbeciles, the insane, the feeble-minded, and habitual criminals. The Legislatures of two States have Bills containing similar provisions now before them, and it is the hope of the Boards of Health in those States that these Bills will pass into law. In nine States Bills of the nature referred to have been before the Legislature and have either been defeated or shelved for an indefinite period. While in the thirty-one remaining States no measures of such a nature have ever been introduced.

As to the method of sterilisation, it is not necessary to give a full medical description. One method is known as vasectomy. The subject of the operation still retains sexual power and desire, and the operation does not cause any change whatever in the sexual organs or interfere with the health or pleasure of the individual. Indeed, the operation is a simple one and quite free from danger, though a little more difficult to perform on the female than on the male. Under the laws of the five States mentioned over 1,000 men have been sterilised and about 100 women.

Of course these laws are too recent—the earliest of them, that of the State of California, was only passed in 1909—to have as yet any measurable effect on general social conditions. It is, therefore, premature to draw conclusions, but it is in order to proffer a word of criticism and caution. The Legislative experiment outlined in the foregoing narrative is in the main not the handiwork of the statesman or jurist, and laws cannot be too much subjected to the rude breath of the populace and the conservative common sense of the man in the street. In the next place, sterilisation is one of the modern short-cuts to Utopia, and short-cuts rarely succeed. With regard to the suggestion that possibly the laws referred to have been passed without due inquiry, it is relevant to mention that when a Bill providing for sterilisation was brought before the Commonwealth of Massachusetts the Legislature appointed a Commission to investigate the alleged increase of criminals, defectives, and degenerates, and the Commissioners reported that in that State crime, degeneracy, imbecility, and insanity were on the wane rather than on the increase. After examining various suggested remedies they found against sterilisation, being of opinion that it would have a tendency to increase sexual vice and spread venereal diseases. And the deliberate and final conclusion of that body of Commissioners was that in their opinion sterilisation had not been demonstrated to be an efficient substitute for segregation and institutional control of confirmed criminals and defectives.

